# Effectiveness of COVID-19 vaccines against ICU admission during Omicron surge in Saudi Arabia: a nationwide retrospective cohort study

**DOI:** 10.1186/s12879-023-08686-y

**Published:** 2023-10-31

**Authors:** Shaymah Aldawish, Raghib Abusaris, Emad Almohammadi, Faten Althobiti, Ahmed Albarrag

**Affiliations:** 1 Department of Epidemiology and Biostatistics, College of Public Health and Health Informatics, King Saud Bin Abdulaziz University for Health Sciences, Riyadh, Saudi Arabia; 2 Public Health Authority, Riyadh, Saudi Arabia; 3 King Abdullah International Medical Research Center (KAIMARC), Riyadh, Saudi Arabia; 4 Collage of Medicine, King Saud University, Riyadh, Saudi Arabia

**Keywords:** Covid-19, ICU admission, Omicron variant, Vaccines

## Abstract

**Introduction:**

Severe acute respiratory syndrome coronavirus 2 (SARS-CoV-2) caused significant economic damage and forced a slew of limitations to be placed by regulatory bodies worldwide. As the SARS-CoV-2 virus continuously mutates over time, it’s crucial to understand how well the vaccines are effective against a new variant.

**Objectives:**

To measure COVID-19 vaccine effectiveness against ICU admission with the Omicron variant in Saudi Arabia regions.

**Methods and materials:**

A retrospective cohort study was conducted of vaccinated and non-vaccinated individuals who tested positive during Omicron dominant period (Jan 1, 2020- Jun 11, 2022). We used a Cox proportional hazards model based on calendar time to assess the vaccine’s effectiveness while controlling for age and gender.

**Results:**

A total of 14103 individuals who were divided into fully vaccinated included 8388 (59.5%) individuals, partially vaccinated included 1851 (13.5%) individuals, and un-vaccinated included 3864 (27.4%) individuals. Higher age was associated with a higher risk of ICU admission (HR = 1.03, 95% CI: 1.02, 1.04). Three doses are associated with a lower risk of ICU admission compared to the single dose (HR = 0.09, 95% CI: 0.04, 0.20). By studying the distribution of Omicron infection among different regions, Al-Madinah Al-Monawarah had the highest proportion at 60.23 per 100,000 population (95% CI: 57.05, 63.53). In contrast, Al-jouf had the lowest proportion at 4.51 per 100,000 population (95%CI: 2.891, 6.713). The vaccination status was significantly different in different regions, as the highest proportion of fully vaccinated participants inhabited in Tabouk region, with 71.8% of its cases. Out of all regions, Najran had the highest proportion of ICU admission among Omicron cases with 20% (95% CI: 9.94%, 34.22%). While the lowest rates existed in Riyadh with 0.86% (95%CI: 0.61%, 1.17%).

**Conclusion:**

We found that a booster significantly enhanced protection against severe COVID-19. The partially vaccinated and unvaccinated participants were at significantly higher risk of ICU admission when compared to the fully vaccinated participants. Furthermore, in future, it is worth investigating the effectiveness of a booster when other potential factors (e.g., region, comorbidities, etc.) are included, particularly among future variants of COVID-19.

## Background

Despite the numerous government interventions, the emergence of the Omicron variant created a significant increase in the number of people infected with COVID-19, more than had been reported with all other variants [1]. On November 9, 2021, Omicron (B.1.1.529) was discovered for the first time in South Africa. It was immediately designated as the fifth variant of concern (VOC). Omicron has a large number of mutations in the spike region, and the appearance of this variant poses a great challenge worldwide because of its high transmission rate and number of mutations [2–4].

The current COVID-19 vaccines were made from an ancestral SARSCoV-2 virus strain. There are considerable concerns about the protective effect of these currently available vaccines against the Omicron variant that is antigenicity distinct from its ancestor virus. According to laboratory data, vaccinated individuals had a significantly reduced neutralizing antibody response to the Omicron variant compared to the original Delta (B.1.617.2) variant, although the booster increased the response to the Omicron variant [5–8]. Early laboratory findings suggest that the original vaccines have a lower vaccine effectiveness (VE) against the Omicron variant infection, as the level of neutralizing antibodies is correlated with the protection against reinfection and with the effectiveness of the vaccine [9, 10].

Studies conducted on the ChAdOx1 nCoV-19 (AstraZeneca) vaccine and the BNT162b2 (Pfizer) vaccine showed that primary immunization with two doses of either vaccine has limited protection against infection and symptomatic disease in individuals infected with the Omicron variant. However, after initial vaccination with ChAdOx1 nCoV19 vaccine or BNT162b2 vaccine, administration of a booster dose of the BNT162b2 vaccine or mRNA-1273 (Moderna) vaccine has been shown to provide some protection, but this protection has decreased over time [11, 12]. Additionally, assessing VE against omicron hospitalization has become more challenging due to the attenuated intrinsic severity and its high prevalence of infection [13].

Previous findings found that there is a reduction in VE of two doses of mRNA vaccines, including mRNA-1273 (Moderna) and BNT162b2 (Pfizer), against infection with BA.1 omicron subvariant compared with earlier VOCs12. However, another study shows that the VE of three doses against hospitalization with BA.1 was initially higher, but its waned quickly. Of concern, the VE of BA.4 and BA.5 Omicron subvariant was found to be decreased compared to BA.1 subvariant [14–17].

In December 2020, Saudi Arabia approved BNT162b2 (Pfizer) as the country's first vaccine. Saudi Arabia later approved the ChAdox1 nCoV-19 of the Oxford-AstraZeneca in February 2021 [18]. Since the approval of these vaccines happened on short notice and happened speedily to prevent the spread of new COVID-19 variants, there is an inherent need to research the efficiency of different vaccines continuously and whether they can still be effectively used against the other variants. A published study found VE was lower against Omicron infection symptoms than Delta. The effectiveness declined by 36% after two months of the second dose and then turned to 1% after six months of the second dose. However, the booster dose increased the effectiveness to 61% after seven days [19].

Clinical studies in Saudi Arabia described therapy and admission in intensive care units, clinical features, and the epidemiology of COVID-19 [20–22]. Most of these studies were conducted earlier, leaving clear research gaps in other waves and the emergence of the Omicron variant [23, 24].

Some researchers have shown that “the available seroprevalence of COVID-19 studies indicate pockets of infection among the certain population and certain provinces” in Saudi Arabia [25]. Overall, findings from this research will help healthcare officials to detect strengths, weaknesses, and potential areas for improvement in the equitable distribution of vaccines, training competency for the vaccination program, and overcoming obstacles that prevent the effective administration of vaccines.

This research aims to investigate the effectiveness of the Covid-19 vaccines against ICU hospitalization during the Omicron Surge in Saudi Arabia.

## Methods & materials

### Study population and design

This retrospective cohort study was conducted in Saudi Arabia from the period of January to June 11, 2022, during the surge of the Omicron variant.

#### Inclusion and exclusion criteria

The study included COVID-19 patients from different region in Saudi Arbia who had positive tests by real-time reverse transcription-polymerase chain reaction (RT-PCR) assay of a nasopharyngeal swab.

Persons were excluded if they received a vaccine other than the two primary COVID-19 vaccines (Pfizer and AstraZeneca) used by the health system. Suspected cases without confirmed results and patients below the age of 18 were excluded from the study. The main analysis was limited to patients with specimens sequenced as part of national sentinel surveillance.

### Data collection

The data were extracted from Public Health Authority (PHA) with referring to the national program for COVID-19 Genomic surveillance, Health Electronic Surveillance Network (HESN) and National Vaccine Registry (NVR). The genetic sequencing was implemented at PHA as part of an initiative to monitor the national genomic epidemiology of SARS-CoV-2 across the country. The sequencing protocol was done following manufacture protocol. The test was done using the RealStar® SARS-CoV-2 RT-PCR Kit (Altona Diagnostics) and the BGI Real-Time Fluorescent RT-PCR kit (BGI). The study population and main analysis were restricted to the proportion of cases with viral genome data for Omicron variant. Collected data included demographic characteristics (e.g., age, sex, residence, nationality), notification information (details of area) and vaccine information (types, number of doses, dates).

### Exposure

The primary exposure was categorized into full vaccination, defined as receiving two doses or more of Pfizer–BioNTech or Oxford– AstraZeneca, and 7 days had passed after receiving the last dose [26].

Individuals were considered partially vaccinated if they had received only one dose and 14 days had passed after the first dose OR if they had received two doses with less than seven days after the second dose [26].

Individuals were considered unvaccinated until receipt of their first dose of Pfizer– BioNTech or Oxford–AstraZeneca or until censoring at disenrollment.
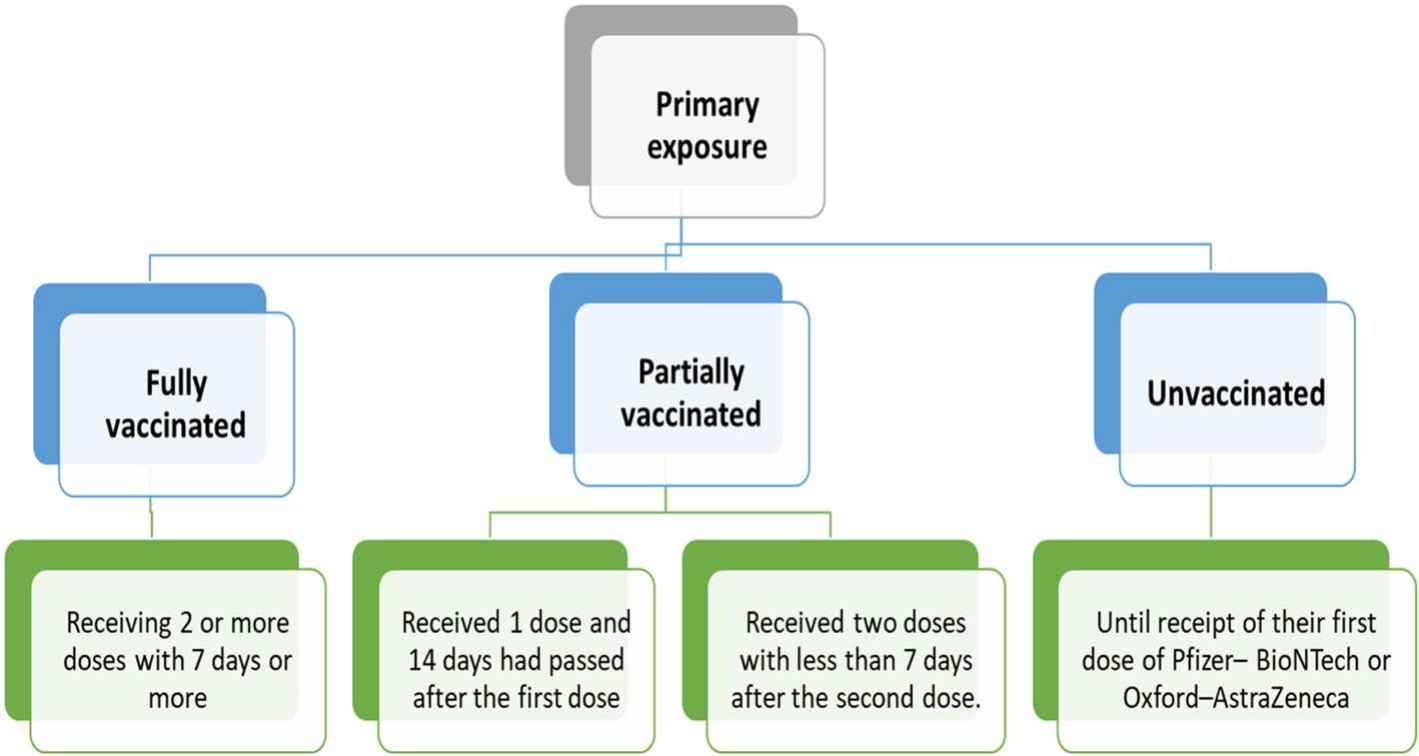


### Outcomes

Vaccine effectiveness was investigated for admission to the intensive care unit (ICU) due to the Omicron variant.

### Sample size

The sample was 14,103 individuals diagnosed with the omicron variant of the SARS-CoV-2 virus. Thus, no sample calculation is needed as well as no sampling technique is required.

### Data management and statistical consideration

Statistical analysis was done by SPSS version 25 [27]. The Kolmogorov–Smirnov normality test was used to test the distribution of quantitative variables to select accordingly the type of statistical testing: parametric or nonparametric. Quantitative non-parametric data were presented as median and interquartile range (IQR) and were analyzed by the Kruskal–Wallis test with Mann Whitney-test for post-hoc testing. Categorical variables were presented as frequency and percentage (%) and analyzed using the Chi-square test.

Binary univariable an multivariable logistic regression was used to study the association of vaccination status with ICU admission while controlling for the other variables. Hazard ratios (HRs) with 95% CIs from the Cox model was calculated.

### Ethics consideration

The research project started after obtaining approval from the Institutional Review Board of PHA in Saudi Arabia (SCDC-IRB-A047-2022). This study proposed no interventions to the participants. Considering the retrospective nature of this study, the requirement for informed consent was waived by Institutional Review Board of PHA. Cases were identified with serial numbers to be anonymous and data storage and access were limited to study investigators only. All methods were carried out in accordance with the ethical principles contained in the declaration of Helsinki (2000) and the Clinical Practice Guidelines.

## Results

This retrospective cohort study was conducted on 14,103 individuals with SARS-CoV-2 who were divided according to vaccination into three groups:

- Fully vaccinated: included 8388 SARS-CoV-2 individuals who had been fully vaccinated by receiving 2 or 3 doses within 7 days or more.
- Partially vaccinated: included 1851 SARS-CoV-2 individuals who had been partially vaccinated subdivided into 1604 patients who received only one dose and 247 who received two doses with less than 7 days after the second dose.
- Unvaccinated: included 3864 SARS-CoV-2 individuals who were not vaccinated.

Table 1 shows the demographics of the studied groups. Kruskal Wallis test was used to compare age, while the Chi-Square test was used for gender and Nationality. Age was significantly different between the studied groups being higher in the vaccinated patients (either fully or partially vaccinated) as compared to the unvaccinated ones (*P* < 0.05). The gender distribution between the studied groups was comparable.

**Table 1:** Demographic data of the studied groups

Variable		Fully vaccinated (*n* = 8388)	Partially vaccinated (*n* = 1851)	Unvaccinated (*n* = 3864)	*P*
Age (years)	**Median (IQR)**	38 (28 – 52)^a^	36 (27 – 51)^b^	33 (17 – 54)^c^	** < 0.001**
Gender	**Male**	3998 (47.7%)	925 (50%)	1908 (49.4%)	0.077
	**Female**	4390 (52.3%)	926 (50%)	1956 (50.6%)	
Nationality	**Saudi**	6627 (79%)^a^	1317 (71.2%)^b^	2347 (60.7%)^c^	** < 0.001**
	**Non-Saudi**	1761 (21%)	534 (28.8%)	1517 (39.3%)	

Data are presented as frequency (%) unless otherwise mentioned, Statistical significance at *P* < 0.05, and different lower-case letters indicate significant difference

In terms of nationality, the vaccinated groups included significantly higher percentages of Saudi participants as compared to the unvaccinated group and the comparison within the vaccinated groups revealed significantly higher percentages of Saudi participants in the fully vaccinated group compared to the partially vaccinated one (*P* < 0.05) (Table 1).

Regarding the outcome of the vaccine exposure, the Chi-square test was used to study the difference in ICU admission in different groups. The vaccinated participants (either fully or partially) were admitted to ICU at a significantly lower rate as compared to the unvaccinated ones *(P* < 0.001). By comparing between the fully and partially vaccinated patients, the fully vaccinated ones had significantly lower rates of ICU admission (*P* < 0.001).

**Fig. 1:**
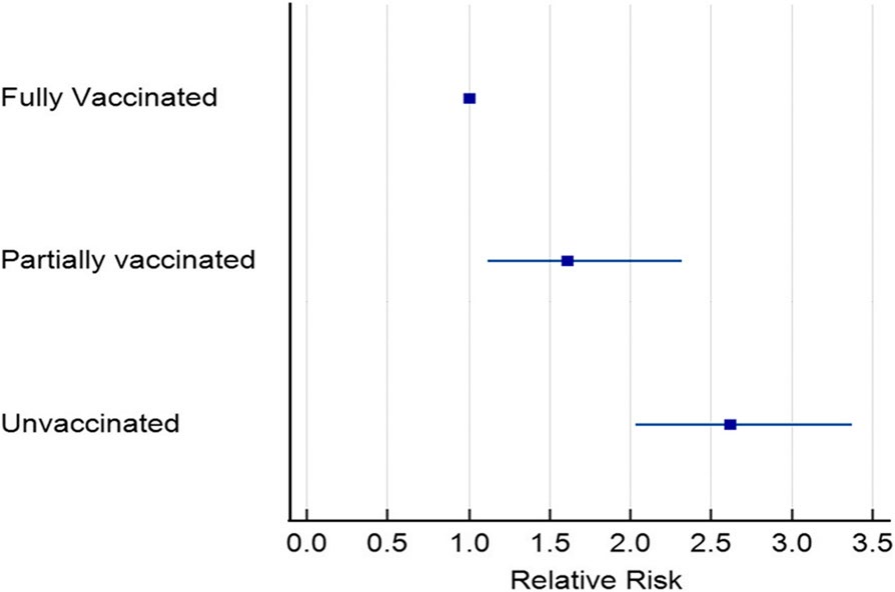
Relative Risk of the outcome of the studied groups

The partially vaccinated and unvaccinated participants were at significantly higher risk of ICU admission [RR (95%CI): 1.609 (1.115: 2.322)] and [RR (95%CI): 2.617 (2.031: 3.372)] respectively when compared to the fully vaccinated participants as shown Table 2, Fig. 1.

**Table 2:** Outcome of the studied groups

		**Fully vaccinated** **(*n* = 8388)**	**Partially vaccinated** **(*n* = 1851)**	**Un-vaccinated** **(*n* = 3864)**	***P***
**ICU admission**	**Yes**	107 (1.3%)^a^	38 (2.1%)^b^	129 (3.3%)^c^	< 0.001*
	**No**	8281 (98.7%)	1813 (97.9%)	3735 (96.7%)	
**Relative Risk** **(95%CI)**			1.609 (1.115: 2.322)	2.617 (2.031: 3.372)	

Data are presented as frequency (%) unless otherwise mentioned, and different lower-case letters indicate significant difference

By studying the distribution of Omicron infection among different regions, Al-Madinah Al-Monawarah had the highest proportion in 60.23 per 100,000 population (95%CI from 57.05 to 63.53) followed by Riyadh region in 48.59 per 100,000 population (95%CI from 47.13 to 50.08) then Makkah Al-Mokarramah region in 43.69 per 100,000 population (95%CI from 42.34 to 45.08) and Eastern region in 32.36 per 100,000 population (95%CI from 30.82 to 33.95). Northern borders, Najran and Al-jouf had the lowest rates of Omicron infection which were, respectively, 6.79 (95%CI from 4.434 to 9.945), 6.57 (95%CI from 4.696 to 8.952) and 4.51 per 100,000 population (95%CI from 2.891 to 6.713) as shown in Table 3**.**

**Table 3:** Distribution of Omicron infection among different regions

	***N***	*Incidence per 100K population*	*95%CI*
**Al-Madinah Al-Monawarah** (*n* = 2,239,923)	1349	60.23	57.05 to 63.53
**Riaydh** (*n* = 8,660,885)	4208	48.59	47.13 to 50.08
**Makkah Al-Mokarramah** (*n* = 9,033,491)	3947	43.69	42.34 to 45.08
**Eastern Region** (*n* = 5,148,598)	1666	32.36	30.82 to 33.95
**Asir** (*n* = 2,308,329)	727	31.49	29.25 to 33.87
**Jazan** (*n* = 1,637,361)	348	21.25	19.08 to 23.61
**Al-Qaseem** (*n* = 1,488,285)	254	17.07	15.03 to 19.3
**Hail** (*n* = 731,147)	103	14.09	11.5 to 17.09
**Al-Baha** (*n* = 497,068)	50	10.06	7.47 to 13.26
**Tabouk** (*n* = 949,612)	85	8.95	7.15 to 11.068
**Northern Borders** (*n* = 383,051)	26	6.79	4.434 to 9.945
**Najran** (*n* = 608,467)	40	6.57	4.696 to 8.952
**Al-jouf** (*n* = 531,952)	24	4.51	2.891 to 6.713

Out of all regions, Najran had the highest incidence of ICU admission among Omicron cases with 20% (95%CI: from 9.94% to 34.22%) followed by Al-Qaseem with 8.66% (95%CI: from 5.67% to 12.59%), then Al-Baha and Al-jouf with 6% (95%CI: from 1.72% to 15.15%) vs. 4.17% (95%CI: from 0.45% to 17.87%) in order while the lowest rates existed in Eastern region with 1.5% (95%CI: from 1.00% to 2.17%), Tabouk with 1.18% (95%CI: from 0.13% to 5.37%) and Riyadh with 0.86% (95%CI: from 0.61% to 1.17%) as shown in Table 4**.**

**Table 4:** Number of ICU admission among Omicron cases in different regions

	**Total**	**ICU admission**	**%**	**95%CI**	
Najran	40	8	20.00%	9.94%	34.22%
Al-Qaseem	254	22	8.66%	5.67%	12.59%
Al-Baha	50	3	6.00%	1.72%	15.15%
Al-jouf	24	1	4.17%	0.45%	17.87%
Jazan	348	14	4.02%	2.32%	6.48%
Al-Madinah	1349	53	3.93%	2.99%	5.07%
Northern Borders	26	1	3.85%	0.42%	16.60%
Hail	103	3	2.91%	0.83%	7.57%
Asir	727	17	2.34%	1.42%	3.63%
Makkah	3947	86	2.18%	1.76%	2.67%
sEastern	1666	25	1.50%	1.00%	2.17%
Tabouk	85	1	1.18%	0.13%	5.37%
Riaydh	4208	36	0.86%	0.61%	1.17%

Data are presented as frequency (%)

The vaccination status was significantly different in different regions as the highest proportion of fully vaccinated participants inhabited the Tabouk region with 71.8% of its population followed by Asir with 64%, then the Eastern region with 62.8%.

Regarding partially vaccinated participants, the highest percentage (22%) inhabited the Al-Baha region, followed by then Hail region with 20.4%. Compared to other regions, most unvaccinated participants (42.1%) lived in Al-Qaseem followed by Najran region with 40%. Table 5

**Table 5:** Distribution of the studied participants according to vaccination status in different regions

	**Fully vaccinated** **(*n* = 8388)**	**Partially vaccinated** **(*n* = 1851)**	**Unvaccinated** **(*n* = 3864)**
Riaydh	2544 (60.5%)	543 (12.9%)	1121 (26.6%)
Makkah Al-Mokarramah	2221 (56.3%)	511 (12.9%)	1215 (30.8%)
Al-Madinah Al-Monawarah	748 (55.4%)	177 (13.1%)	424 (31.4%)
Al-Qaseem	119 (46.9%)	28 (11%)	107 (42.1%)
Eastern	1046 (62.8%)	199 (11.9%)	421 (25.3%)
Asir	465 (64%)	115 (15.8%)	147 (20.2%)
Tabouk	61 (71.8%)	13 (15.3%)	11 (12.9%)
Hail	48 (46.6%)	21 (20.4%)	34 (33%)
Northern Borders	14 (53.8%)	5 (19.2%)	7 (26.9%)
Jazan	211 (60.6%)	59 (17%)	78 (22.4%)
Najran	16 (40%)	8 (20%)	16 (40%)
Al-Baha	27 (54%)	11 (22%)	12 (24%)
Al-jouf	15 (62.5%)	4 (16.7%)	5 (20.8%)
*P* value	** < 0.001**		

Data are presented as frequency (%), Statistical significance at *P* value < 0.05

The binary univariable logistic regression analysis in Table 6 showed that age and vaccine exposures were significantly associated with the incidence of ICU admission.

**Table 6:** Binary univariable and multivariable logistic regression of participants’ characteristics associated with ICU admission

	**Univariable analysis**			**Multivariable analysis**		
	**uOR**	**95%CI**	***P***	**aOR**	**95%CI**	***P***
**Age (years)**						
< 20	Ref			Ref		
20–40	0.415	0.28- 0.614	** < 0.001**	0.527	0.353- 0.786	**0.002**
> 40 years	1.191	0.85- 1.671	0.310	1.481	1.049- 2.091	**0.026**
**Gender**						
Male	Ref			Ref		
Female	0.952	0.75- 1.21	0.689	0.96	0.743–1.239	0.752
**Vaccine exposure**						
Fully vaccinated	Ref			Ref		
Partially vaccinated	1.622	1.116- 2.357	**0.011**	1.539	1.015- 2.334	**0.043**
Unvaccinated	2.673	2.063- 3.464	** < 0.001**	2.749	2.079- 3.635	** < 0.001**

*uOR* Unadjusted Odds ratio, *aOR* Adjusted Odds ratio, Statistical significance at *P* value < 0.05

In regards age, participants aged between 20 and 40 years old had significantly lower odds of being admitted to the ICU as compared to those younger than 20 years old (OR: 0.415, 95%CI: 0.28 to 0.614, *P* < 0.001). Regarding vaccine exposure, the partially vaccinated participants (OR: 1.622, 95%CI: 1.116 to 2.357, *P* = 0.011) and the unvaccinated ones (OR: 2.673, 95%CI: 2.063 to 3.464, *P* < 0.001) had significantly higher odds of ICU admission compared to the fully vaccinated ones.

By studying multivariable logistic regression, age and vaccine status were significantly associated with the incidence of ICU admission as shown in Table 6.

Regarding age, participants in the age group 20–40 had significantly lower odds of being admitted to ICU as compared to those < 20 (OR: 0.527, 95%CI: 0.353 to 0.786, *P* = 0.002), while those older than 40 had higher odds of ICU admission as compared to those < 20 (OR: 1.481, 95%CI: 1.049 to 2.091, *P* = 0.026).

As compared to fully vaccinated participants, the partially vaccinated and the unvaccinated ones had significantly higher odds of ICU admission which were, respectively, (OR: 1.539, 95%CI: 1.015 to 2.334, *P* = 0.043) and (OR: 2.749, 95%CI: 2.079 to 3.635, *P* < 0.001).

**Fig. 2:**
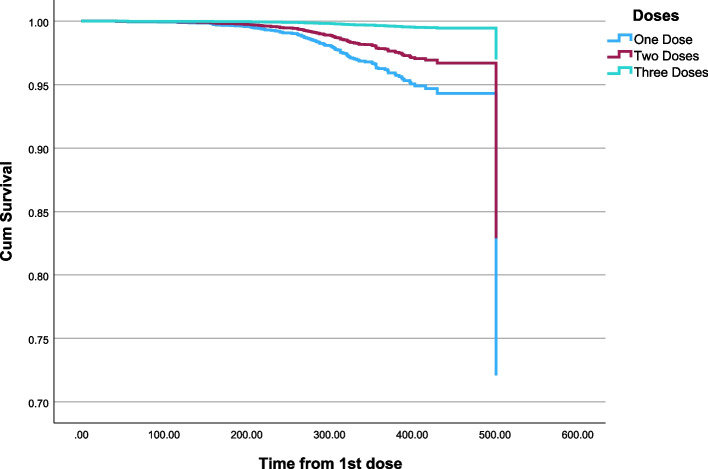
Cox regression for the effect of number of doses on ICU admission controlling for age and gender

Single and multiple Cox regressions for the effect of number of doses on ICU admission controlling for age and gender. Age and dose were statistically significant while gender was not statistically significant. Higher age was associated with a higher risk of ICU admission (HR = 1.03, 95% CI: 1.02, 1.04), *p* < 0.001. While a higher number of doses is associated with a lower risk of ICU admission. HR for two doses as compared to single dose (HR = 0.57, 95% CI: 0.37, 0.89), *p* = 0.013. HR for three doses as compared to a single dose (HR = 0.09, 95% CI: 0.04, 0.20), *p* < 0.001 (Table 7, Fig. 2).

**Table 7:** Cox regression for the effect of the number of doses on ICU admission controlling for age and gender

	**Univariable**			**Multivariable**		
	**HR**	**P**	**95.0% CI for HR**	**HR**	***P***	**95.0% CI for HR**
**Gender**						
** Male**	1.00			1.00		
** Female**	0.88	0.464	0.63- 1.23	0.85	0.387	0.59- 1.23
** Age**	1.03	** < 0.001**	1.02- 1.04	1.03	** < 0.001**	1.02- 1.04
**Doses**						
** Single dose**	1.00			1.00		
** Two doses**	0.57	**0.006**	0.38- 0.85	0.57	**0.013**	0.37- 0.89
** Three doses**	0.10	** < 0.001**	0.05- 0.19	0.09	** < 0.001**	0.04- 0.20

Statistical significance at *P* value < 0.05

## Discussion

The BA.1 subvariant of Omicron was responsible for the initial Omicron outbreaks around the world. However, BA.1 has been quickly replaced by BA.2 within months, and later by BA.4 and BA.5 (BA.4/5). As of early September 2023, the subvariants of Omicron are circulating, including EG.5, XBB.1.5, and XBB.1.16, and are considered the dominant nationwide. Subsequently, these characteristics of Omicron affect how it spreads and responds to treatments and vaccinations [28–30].

In early December 2021, the first case of Omicron was reported in Saudi Arabia with a highly transmissible nature and risk of immune evasion. However, since the beginning of COVID-19 in early March 2020, the government in Saudi Arabia implemented a comprehensive response to prevent the pandemic surge involving travel restrictions, lockdowns of schools and universities, and suspension of attendance, followed by a complete curfew. Moreover, the Umrah was suspended, and the booking of Hajj was restricted to local COVID-19 recovered cases [31–33].

We conducted a cohort study including 14,103 individuals with SARS-CoV-2 living in KSA, aiming to estimate the distribution of Omicron variant in different regions of Saudi Arabia and to determine the effectiveness of different types of vaccines with the Omicron variant. In the current study, 59.48% were fully vaccinated (> 7 days after of two or more doses), 13.12% were partially vaccinated (> 14 days after first dose through day 7 after second dose), and 27.40% were unvaccinated (days from cohort entry until receipt of first vaccine dose) [26]. Unvaccinated individuals were significantly younger than the vaccinated and partially vaccinated population with *p* < 0.001, as well the largest proportion of the unvaccinated group were non-Saudi with *p* < 0.001.

The high coverage of COVID-19 vaccination among the Saudi population is translated to the effort of the government in implementing a ranged distribution plan for vaccination targeting the largest size of the population of each city, prioritizing cities of high population as the capital Riyadh, followed by Jeddah, Dammam, Madinah, and Makkah. [34].

In the present study, simple and multiple logistic regression is used to study the association between vaccination status and ICU admission while controlling for age and gender. In unvaccinated participants were 2.7 times higher of being admitted to the ICU compared to the fully vaccinated participants. This is in line with a study published recently they found hospitalization rates were 10.5 times higher in unvaccinated persons and 2.5 times higher in vaccinated persons with no booster dose [35]. However, some studies found the protection against Omicron depends on the type of vaccine, in a large cohort research in Singapore involving over 2.5 million people aged 30 or older. These data demonstrate that booster mRNA vaccine protection against severe COVID-19 was persistent over six months independent of vaccine combination, and 3-dose of inactivated vaccine type gave more protection than 2-dose but less protection than 3-dose mRNA [36].

Also, Cox regression is conducted to see the effect of number of doses on ICU admission while adjusted for age and gender. We found that the HR for ICU admission is increased when the age is increased. Similar results have been observed in previous literature they found those under 40 years old represent a small proportion of the total number of most severe COVID-19 cases in Europe [37]. Our finding found that there is no difference between males and females in regard to ICU admission. In contrast, an early finding revealed that men are more at risk for a worse outcome [38].

At the same time, the risk of admission to the ICU is decreased with a higher number of doses. This analysis shows that the booster and two doses effectively reduce the risk of ICU admission due to Omicron infection, compared to one dose by 91% and 43%, respectively. This finding is similar to the Qatari study, they found that booster is effective by 76.5% (95% CI, 55.9%-87.5%) against Omicron-related hospitalization and death [16].

We also studied the distribution of the Omicron variant across different regions of Saudi Arabia. The first conducted study for Omicron-infected patients was in a single medical center in Saudi Arabia. This was achieved by AlBahrani et al., showing that the rate of hospitalization (14%) was lower than previously reported in the first and second wave of COVID-19. Nonetheless, the hospitalization rate was inversely correlated with the number of vaccination doses with least admission (5.4%) among fully vaccinated patients. They reported a rate of ICU admission 3.5% and 2% mechanical ventilation rate [1].

In the current study, the vaccination status was significantly different in different regions as the highest proportion of fully vaccinated participants inhabited Tabouk with 71.8% followed by Asir region with 64% then the Eastern region with 62.8% of its population.

Regarding Omicron infection, Al-Madinah Al-Monawarah had the highest number of cases followed by Riyadh region, then Makkah Al-Mokarramah region. It is worth mentioning that during the study period (Jan 2022- Jun 2022), Saudi Arabia has lifted all COVID-19 restrictions on Hajj and Umrah for local and international pilgrims. The announcement was made after the Ministry of Hajj and Umrah released Ramadan 2022 Operational Plan of the two holy mosques [39]. This might explain the highest number of cases, especially in Al-Madinah and Makkah.

The disparity in ICU among regions may include several factors; the literature indicates the association between socio-demographic factors and variations in COVID-19 outcomes. Likewise, many studies have reported the relationship between comorbidities and severe COVID-19 [40–42]. For example, the fact that Najran had the highest rate of ICU admission could relate to advanced age and comorbidities such as Type 2 diabetes mellitus (DM2), cardiovascular disease, and obesity, which was discussed previously in a national study [43]. Whereas demographics factors and comorbidities are related to regional variation, other factors, such as disparities in income, access to healthcare resources, education levels, and overall population health, are associated with the COVID-19 outcome in different regions [44–46].

### Limitations

One of the limitations in this study is the data was only limited to the samples received by PHA as a part of surveillance. Also, the assessment of differences in behavior or adherence to the COVID-19 precautions are unaccounted among vaccination groups in this study. For example, those who were unvaccinated may be less likely to wear a mask or take precautions. So, this could either lead to overestimation or underestimation. However, this limitation is minimized because of the high willingness and rate of vaccination in Saudi Arabia.

To eliminate confounders, we adjusted for age and sex, but we did not account for other factors that may have influenced the outcomes, such as comorbidity, obesity, smoking and occupation. However, given the study’s observational nature, residual confounding remains possible despite adjustment for several potential confounders.

We did not estimate the vaccine's effectiveness against death, symptomatic infections or organ injury because we assessed only patients who have been admitted to ICU.

### Strengths

To the best of our knowledge, this study is the first investigation to analyze and report the effectiveness of two different vaccines against the COVID-19 Omicron variant in Saudi Arabia. Nonetheless, this study includes a large and diverse population from various regions in Saudi Arabia. As the majority of all ages had already received their third doses during Omicron dominant period, it was possible to estimate the effectiveness of two and three doses in the study period.

## Conclusions

The third dose significantly enhanced protection against severe COVID-19. Also, the partially vaccinated and unvaccinated participants were at significantly higher risk of ICU admission when compared to the fully vaccinated participants. This result supports the earlier studies that the need for booster doses to increase protection against severe COVID-19 outcomes. As the SARS-CoV-19 mutates rapidly and in response to any further SARS-CoV-2 variants, our analysis highlights the necessity of continued research and monitoring of vaccine effectiveness over time to inform policy.

## Data Availability

The data may be provided on reasonable request to the corresponding author.
